# COVID-19 Vaccine Misperceptions in a Community Sample of Adults Aged 18–49 Years in Australia

**DOI:** 10.3390/ijerph19116883

**Published:** 2022-06-04

**Authors:** Kristen Pickles, Tessa Copp, Gideon Meyerowitz-Katz, Rachael H. Dodd, Carissa Bonner, Brooke Nickel, Maryke S. Steffens, Holly Seale, Erin Cvejic, Melody Taba, Brian Chau, Kirsten J. McCaffery

**Affiliations:** 1 Faculty of Medicine and Health, Sydney Health Literacy Lab, School of Public Health, The University of Sydney, Sydney 2006, Australia; tessa.copp@sydney.edu.au (T.C.); rachael.dodd@sydney.edu.au (R.H.D.); carissa.bonner@sydney.edu.au (C.B.); brooke.nickel@sydney.edu.au (B.N.); erin.cvejic@sydney.edu.au (E.C.); melody.taba@sydney.edu.au (M.T.); bcha0834@uni.sydney.edu.au (B.C.); kirsten.mccaffery@sydney.edu.au (K.J.M.); 2 Population Wellbeing and Environment Research Lab, School of Health and Society, University of Wollongong, Wollongong 2522, Australia; gideon.meyerowitzkatz@health.nsw.gov.au; 3 Western Sydney Diabetes, Western Sydney Local Health District, Sydney 2006, Australia; 4 National Centre for Immunisation Research and Surveillance, Kids Research, Sydney Children’s Hospital Network, Sydney 2006, Australia; maryke.steffens@health.nsw.gov.au; 5 School of Population Health, Faculty of Medicine and Health, The University of New South Wales, Sydney 2006, Australia; h.seale@unsw.edu.au

**Keywords:** COVID-19, vaccination, vaccines, misinformation, beliefs, misperceptions, vaccination willingness, vaccine uptake, vaccine knowledge, trust

## Abstract

Central to a successful population vaccination program is high uptake of vaccines. However, COVID-19 vaccine uptake may be impeded by beliefs based on misinformation. We sought to understand the prevalence and nature of misbeliefs about COVID-19 vaccines, and identify associated factors, shortly after commencement of Australia’s national vaccine rollout. A cross-sectional survey was administered to unvaccinated young adults (n = 2050) in Australia aged 18–49 years (mean age 33 years), 13 July–21 August 2021. This sample was previously under-represented in COVID-19 research but shown to have less willingness to vaccinate. Two thirds of participants agreed with at least one misbelief item. Misperceptions about COVID-19 vaccines were found to be significantly associated with lower health literacy, less knowledge about vaccines, lower perceived personal risk of COVID-19, greater endorsement of conspiracy beliefs, and lower confidence and trust in government and scientific institutions. Misbeliefs were more common in participants with less educational attainment, in younger age groups, and in males, as per previous research. Understanding determinants and barriers to vaccination uptake, such as knowledge and beliefs based on misinformation, can help to shape effective public health communication and inform debunking efforts at this critical time and in the future.

## 1. Introduction

The COVID-19 pandemic has prompted the largest vaccination program in history [1]. Central to a successful population vaccination program is high uptake of vaccines. Longitudinal surveys of adults in Australia conducted prior to the availability of COVID-19 vaccines and when case numbers of the virus were low (April–July 2020) showed high rates of vaccination willingness (86–90% willing) [2,3]. However, younger adults perceived themselves to be at lower risk of infection and were less willing to receive a vaccine [4]. This has also been shown internationally [5]. Common concerns about COVID-19 vaccines include vaccine safety, side effects, and long-term effects [3,6,7]. Access barriers to vaccination such as cost and local availability have also been identified as influencing COVID-19 vaccine uptake internationally [8] and vaccination willingness among younger adults [9].

Misinformation about vaccines has been shown to negatively influence people’s attitudes and intentions towards vaccination, such as in the context of human papillomavirus (HPV) and measles-mumps-rubella (MMR) vaccines [10]. Several studies now suggest that COVID-19 vaccine uptake may be impeded by beliefs based on misinformation [11,12,13]. For example, Loomba et al. reported a 6.2–6.4% point decline in vaccination intention among people in the UK and US following exposure to misinformation [11]. It has also been shown globally that certain sociodemographic groups have a greater tendency to believe misinformation (e.g., [14]). In Australia, 13–17% of 4362 adult respondents to a national survey indicated agreement with various forms of misinformation about COVID-19 in 2020 [15]. Lower trust in institutions and greater rejection of official accounts were associated with stronger misbeliefs. Another key finding of this study was that agreement with misinformation was significantly associated with younger age. This survey was conducted many months before COVID-19 vaccines were approved in Australia and large-scale COVID outbreaks (Delta and Omicron) that have since occurred nationally.

At the time of the initial vaccination rollout in Australia, little was known or had been published on public knowledge and beliefs about COVID-19 vaccines. Some prior research demonstrated an association between vaccine hesitancy or refusal and having inadequate knowledge about COVID-19 [2,6]. As these studies were conducted prior to widespread vaccine rollout, vaccine intentions were hypothetical, and knowledge measures were based on knowledge of recommended actions to protect oneself from contracting COVID-19 rather than vaccine-specific knowledge. The current study used a validated measure of vaccine knowledge to determine whether people’s knowledge of vaccines was associated with the likelihood of them holding misbeliefs about COVID vaccines.

In this study, we sought to understand the prevalence and nature of beliefs about vaccines generally and COVID-19 vaccines specifically, and factors associated with vaccine misbeliefs, shortly after the commencement of the Australian national vaccine rollout. Our target population comprised unvaccinated younger adults (18–49 years), as this sample was under-represented in COVID-19 research but shown to have less willingness to vaccinate [6]. Understanding determinants of and barriers to vaccination uptake, such as knowledge and beliefs based on misinformation, can help to shape effective public health communication and inform debunking efforts at this critical time and in the future.

## 2. Methods

### 2.1. Study Design

A national cross-sectional online survey built and administered using the Qualtrics (SAP SE) online survey platform. The study was approved by the University of Sydney Human Research Ethics Committee (2020/212).

### 2.2. Setting

The survey was distributed nationally with data collected between 13 July to 21 August 2021. The highly infectious SARS-CoV-2 Delta variant (B.1.617.2) of COVID-19 was first detected in Australia in June 2021. In response to this outbreak, almost half of Australia’s population and most major cities were not permitted under public health law to leave their place of residence (with exemptions) from early July 2021, and new record daily cases in Australia were recorded in August 2021 [16]. The states of New South Wales and Victoria were disproportionately impacted by local outbreaks [17]. Until this point, COVID-19 had been relatively well controlled by Australia’s strong public health measures, so this was a new experience of risk exposure for the Australian community.

Australia’s nationwide COVID-19 vaccination program began on 21 February 2021. At the time of the survey, 10.2–19.2% (13 July–21 August) of the adult population aged 16–49 years were fully vaccinated against COVID-19 (i.e., had received two doses of vaccine), and 15.5–34.4% (13 July–21 August) over 16 years had received at least one dose [18]. We have reported elsewhere that in July–August 2021, 43% of adults 18–49 years intended to receive a vaccine as soon as possible, with 6% responding that they would never get a COVID-19 vaccine [9].

### 2.3. Participants

Participants were aged 18 to 49 years, able to read and understand English, currently residing in Australia, and had not yet received a COVID-19 vaccine. Individuals who had received any dose of a COVID-19 vaccine were excluded. We recruited participants through Taverner Research, an Australian-owned market research company, using an online panel with approximately 1 million panel members aged under 50 years. Panel members were sent an email invitation to participate in the study and received points for completing the survey, which they could redeem for a range of rewards including gift cards, cash, and vouchers.

### 2.4. Measures

Relevant measures for this analysis are detailed in Table 1. Sociodemographic variables collected included age, education, gender, and residential state. Health literacy was assessed using a single item literacy screener [19]. Digital health literacy was assessed using the eHealth Literacy Scale [20].

For the main outcome measure—COVID-19 vaccine related misperceptions, a list of items was curated from international official health myth buster websites [21,22,23], published literature and mainstream media and commonly reported myths.

We also measured general vaccine knowledge using a validated scale [24], acceptance of vaccine conspiracies, perceived risk of COVID-19, and confidence and trust in government and institutions.

Participants were asked whether they had encountered any negative information about COVID-19 vaccines, and if so, where. They were also asked to briefly describe any content discouraging people from vaccinating that they had come across in the past week.

### 2.5. Statistical Analysis

Analyses were conducted using Stata/IC (v16.1; StataCorp LLC, College Station, TX, USA). The threshold for statistical significance was set at *p* < 0.05 (2-tailed). Descriptive statistics (means and SD for continuous variables, and frequency and relative frequency for categorical variables) were calculated for participant characteristics and study outcomes.

Multivariable truncated linear regression analyses of mean vaccine misbeliefs were conducted, controlling for age, gender, educational attainment, and state of residence. Truncation was based on possible values of the derived outcome variable (i.e., lower bound of 1 and an upper bound of 5). A base model was first constructed including only the control variables listed above, and then, as a full model with all potential explanatory variables included (see Table 2). *p*-values less than 0.05 were considered statistically significant. Estimates are provided to 3 significant figures.

Responses to all misbelief items at baseline were moderately correlated (pairwise Rs between 0.21–0.64), with good internal consistency (Cronbach’s alpha = 0.9).

Qualitative data (free text responses in the survey) were analysed using content analysis [31]. BC and MT read through all responses to familiarize themselves with the content and generated a preliminary list of recurring themes. These were discussed with RD, TC and KP. BC and MT then finalized the coding framework and applied it to the entire dataset. Discrepancies were discussed and resolved at team meetings. We assessed level of agreement between the reviewers using the Cohen kappa statistic, which indicated high agreement (k = 0.968). Descriptive statistics were calculated to summarise the frequency of occurrence of each code. Illustrative quotes are presented in the results to highlight each theme.

## 3. Results

### Sample Characteristics

Characteristics of the 2050 participants are summarised in Table 2. Mean age of participants was 33 years, 42.3% (n = 868) held a university degree, 83.3% (n = 1707) were classified as having adequate health literacy, and most of the sample resided in New South Wales (30.2%; n = 619), Victoria (25%; n = 513), or Queensland (21.4%; n = 439) (Australia’s three most populous states). Our sample had slightly higher educational attainment when compared to national data (42.3% versus 37.8% university degree) but was otherwise comparable to national estimates [32].

## 4. Confidence and Trust in Vaccines

The majority of participants had high trust in the safety (87.9%) and effectiveness (85.8%) of childhood vaccines and in flu (safety 79.2%; effectiveness 75%) and travel vaccines (safety 73.7%; effectiveness 73.5%) (Table 3). Two-thirds (~65%) of participants trusted the safety and effectiveness of the Pfizer vaccine, while a lower proportion (~40%) indicated the same trust for the AstraZeneca vaccine.

Regarding confidence in COVID-19 vaccine benefits, 70.5% thought that getting a COVID-19 vaccine will protect other people in their community, 66.3% said that a COVID-19 vaccine will be moderately or very safe, 62.5% of participants indicated that getting a COVID-19 vaccine will be important for their health, and 47.6% were moderately or very concerned that a COVID-19 vaccine would cause them to have a serious reaction.

## 5. Misperceptions, Conspiracy Beliefs, and Vaccine Knowledge

Table 4 shows mean scores for the COVID-19 vaccine misbelief and acceptance of vaccine conspiracy items, and percentage correct for the vaccine knowledge items.

### 5.1. COVID-19 Vaccine Misperceptions

Overall, 79.4% (n = 1628) of participants had a mean score of less than 3/5 on the vaccine misbelief items, indicating an average disagreement with the vaccine misbelief items, and 10.6% (n = 218) strongly disagreed with all items. Sixty-six percent (n = 1352) of participants scored higher than 3 on at least one item, indicating at least one misbelief.

Table 5 shows the most common COVID-19 vaccine misperceptions were: COVID vaccinated people can affect non-vaccinated people’s health (agreed: n = 602, 29.4%), people who have had a COVID-19 vaccine shed the virus to others (agreed: n = 459, 22.4%), and COVID-19 vaccines cause immune damage (agreed: n = 386, 18.8%). Seven hundred twenty-one (35.2%) participants were unsure regarding whether COVID-19 vaccines have been linked to infertility.

General anti-vaccination beliefs were held by some participants; 12.6% (n = 258) of the sample indicated “true” that “vaccines cause autism”, with 61.9% (n = 1269) stating that this statement is false. A 25.5% share of participants (n = 523) were unsure.

### 5.2. Vaccine-Related Conspiracy Beliefs

As shown in Table 6, 651 (31.8%) participants agreed with the statement “people are deceived about the effectiveness of vaccines”. Additionally, 607 (29.6%) agreed that people are deceived about vaccine safety, and 571 (27.9%) agreed that drug companies cover up the dangers of vaccines.

### 5.3. Vaccine Knowledge

On average, participants scored correctly on 49% (~4 of the 8 vaccine knowledge questions). As shown in Table 7, most participants scored correctly on the following three items: “the effectiveness of vaccines has been proven” (Agree: n = 1629; 79.5%), “vaccines are not needed because diseases can be treated” (Disagree: n = 1619; 79.0%), and “without broadly applied vaccine programs, smallpox would still exist” (Agree: n = 1507; 73.5%).

Participants were more likely to have incorrect knowledge about vaccine safety (“the doses of chemicals used in vaccines are not dangerous for humans” (Agree: n = 504; 24.6%)), vaccines and the immune system (“the immune system is overloaded if we are given too many vaccinations” (Disagree: n = 473; 23.1%)), and disease resistance (“people would be more resistant to diseases if they were not given so many vaccines” (Disagree: n = 384; 18.7%)).

Regarding uncertain responses, 946 (46.1%) of participants did not know whether vaccinations increase the occurrence of allergies. There was also uncertainty in response to questions about vaccine safety and side effects.

### 5.4. Factors Associated with Greater Agreement with COVID-19 Vaccine Misbeliefs

Estimated marginal mean values from the multivariable regression model of COVID-19 vaccine misbeliefs are provided in Table 8. Greater agreement with COVID-19 vaccine misbeliefs was significantly associated with younger age (*p* < 0.01), male gender (*p* < 0.01), and lower educational attainment (*p* < 0.001).

After controlling for age, gender, education, and state of residence, misperceptions about COVID-19 vaccines were found to be significantly associated with lower health literacy, less knowledge about vaccines, lower perceived personal risk of COVID-19, greater endorsement of non-COVID conspiracy beliefs, lower confidence in government, and lower trust in scientific institutions.

The only control variable that remained significantly associated with COVID-19 vaccine misperceptions in the adjusted model was lower education, while age and gender became non-significant. There were no differences observed between states in any model.

### 5.5. Content Discouraging People from Having a COVID-19 Vaccine

A total of 963 participants (47% of sample) responded to the free-text question: “In the last week have you come across any content discouraging people from vaccinating? (i.e., on your social media, from friends, at the workplace)?”. Seventeen themes were developed which captured the type of discouraging content participants witnessed (Table S1). The three most reported themes are summarised below, and the top 5 themes are shown in Table 9, with example quotes.

1. *Negative vaccine side effects and vaccine risk* (n = 384, 39.9%): Included responses pertaining to concerns about specific and general side effects of the COVID-19 vaccine as well as concerns about adverse events post-vaccination and overall safety of the vaccine. Specific side effects of concern included blood clots, fertility and reproductive concerns, motor, cognitive and neurological disorders, AstraZeneca-specific concerns, shaking, paralysis, pain, fever and fainting.
2. *Conspiracy theories* (n = 133, 13.8%): Example conspiracy theories commonly raised included virus legitimacy scepticism, 5G, tracking, microchips, magnetism caused by the vaccine and vaccine causing COVID-19 or other disease.
3. *Antivax or hesitance towards vaccine* (n = 115, 11.9%): Responses included general anti-vaccination sentiments (e.g., “strongly against vaccine for well-informed reasons” or “not liking vaccines”), reluctance to receive vaccinations and mentions of “Antivaxxers” as community.

Of the 963 participants, 268 (27.8%) also reported the source of the content discouraging vaccination. From these, the majority (n = 182; 67.9%) reported the source to be social media, with commonly reported platforms including Facebook, Instagram and TikTok. Family, friends and colleagues were the next most frequently mentioned source (n = 57; 21.3%), followed by traditional media (n = 46; 17.2%) (Table S2).

## 6. Discussion

This analysis showed that one in five younger adults aged 18–49 years in Australia on average agreed with some items of misinformation about COVID-19 vaccines, with two thirds indicating agreement with at least one misbelief item. Misperceptions about COVID-19 vaccines were found to be significantly associated with lower health literacy, less knowledge about vaccines, lower perceived personal risk of COVID-19, greater endorsement of non-COVID conspiracy beliefs, lower confidence in government, and lower trust in scientific institutions. Misbeliefs were more common in participants with less educational attainment, in younger age groups, and in males. These findings are similar to those of our earlier COVID misinformation study [15]; however, it comprised an older age cohort (age range 18–90 years; mean age 43 years) versus the younger sample of the current study (age range 18–49 years; mean age 33 years). The results also replicate international studies that have reported on predictors of susceptibility to misinformation (e.g., [14]); however, these studies did not examine predictors of vaccine-specific misinformation.

The most common COVID-19 vaccine misperceptions in this study were perceptions that COVID vaccinated people can affect non-vaccinated people’s health (refer to footnote in Table 5), shed the virus to others, and that COVID-19 vaccines cause immune damage. Uncertainty was also notably high regarding whether COVID-19 vaccines have been linked to infertility. Isolating specific misbeliefs about COVID-19 vaccines can support communication experts and public health agencies to better target their messages to address vaccine misunderstandings. Such misperceptions are relatively common in the broader vaccine context; for example, 21% of Australian parents surveyed in 2017 believed that vaccines can cause autism [27].

While around 20% of our sample on average agreed or strongly agreed with misbelief items, this is of concern, given that increased susceptibility to misbeliefs has been found to reduce self-reported compliance with COVID prevention measures including intention to get vaccinated, likelihood of recommending COVID-19 vaccines to family and friends [11,14], and confidence in the safety of COVID-19 vaccines [12]. Uptake of COVID-19 booster injections (i.e., additional vaccine doses after primary vaccinations) has been low relative to initially high vaccine uptake in Australia [33]. It will be imperative to prioritise correcting misperceptions about COVID-19 vaccines such as those identified in this study to achieve higher rates of booster injections. It is also important to recognise that continued success in COVID-19 and other vaccination programs relies on general community support, which may be harder to gain if one in five people hold misbeliefs about COVID-19 vaccines.

Vaccine misperceptions were more common in those with lower health literacy and educational attainment. These groups have also been shown to be less willing to receive a COVID vaccine [34]. Understanding and critically evaluating health information is a fundamental challenge for a large proportion of the community [35,36], and especially in the context of the COVID-19 infodemic [37,38]. Our study helps to pinpoint specific beliefs about vaccines that can be addressed in health literate-sensitive formats as a starting point, with an overall aim to improve health literacy of populations in need of information about COVID vaccines and in those providing that information.

Participant scores on the vaccine knowledge scale were relatively low but similar to other studies that have used this validated measure (e.g., [39]). This warrants further investigation in younger populations given that those with lower knowledge of vaccines generally showed more misbeliefs about COVID-19 vaccines, and have demonstrated less likelihood of vaccination [39]. Our results highlight specific gaps in our communications about vaccines generally, and specifically about safe vaccine doses, immune system overload, and vaccine resistance. Targeting vaccine literacy may be beneficial in countering vaccine misperceptions [40]. However, vaccine beliefs are complex, and people may have misperceptions about vaccines for a variety of reasons, not simply due to insufficient knowledge.

We identified conspiracy beliefs particularly relating to the public being deceived about safety and effectiveness of vaccines, with stronger endorsement of deception beliefs associated with higher likelihood of holding misbeliefs. A 2020 US-based study reported that respondents who doubted the safety of COVID-19 vaccines had greater likelihood of having conspiracy beliefs and lower vaccine knowledge [39]. Other studies have reported an association between conspiracy beliefs about COVID and propensity to reject information from expert authorities [41], and reduced likelihood of following public health guidance [42,43,44], which can have negative consequences such as vaccine refusal.

Higher trust in scientific institutions and confidence in the government were associated with decreased susceptibility to misbeliefs about COVID-19 vaccines. Trust and confidence in medical and scientific experts, and government, have also been shown to be strong predictors of vaccine acceptance across countries and compliance with public health advice [45,46,47]. Trust in government and institutions was low in this study compared to our previous sample [15], which has been demonstrated in other studies more broadly in younger age samples, particularly in the context of confidence in political institutions (e.g., [48]).

Trust has been central in the context of COVID-19 and COVID-19 vaccines, as there has been, at times, significant uncertainty and incomplete evidence and information given the rapid speed with which the pandemic has progressed, and vaccine development response. In the first six months of vaccine rollout, Australia experienced delays in accessing vaccines produced overseas and uncoordinated delivery, which contributed to missed vaccine targets [49]. There was also vaccine hesitancy due to safety concerns over the AstraZeneca vaccine, which drew significant negative publicity; despite the low risk, people developed preferences for specific vaccines that were difficult to shift [50]. Our participants had more confidence in the safety and effectiveness of other vaccines such as travel and childhood vaccines than COVID-19 vaccines, indicating that there may be factors unique to COVID vaccines that determine people’s acceptance and reactions.

Confidence and trust in vaccine benefits and safety may be enhanced by fostering trust more broadly in the healthcare system, science, and government [51]. It is important to understand the role of trust in relation to vaccine misperceptions so that communications and interventions can be specifically designed and implemented in a way that will reach and appeal to groups that are distrustful of authorities developing, dispensing, and communicating about COVID vaccines. Interventions to strengthen public trust in science, increase information literacy, and combat misinformation are as important as ever.

## 7. Limitations

Population attitudes, motivations, and beliefs change rapidly and will likely have fluctuated throughout the various stages of the pandemic in response to public health campaigns and advice, vaccine availability and access, and community risk. It is important to regularly track factors that are associated with vaccination willingness to continue to inform communications and to ensure that public health campaigns are responsive to on-the-ground drivers of vaccine uptake.

This study was conducted in a high-income country at a single point in time, in the context of rapidly changing pandemic risk, information, and vaccination availability and rollout. The survey was distributed pre-vaccine rollout for this age group, so responses may have changed over time. Trust, for example, may have shifted as more information about vaccine safety and efficacy became available. Our measure of vaccine knowledge was validated but not specific to COVID-19 vaccines; this may be explored in future studies. Our measure of COVID-19 vaccine misbeliefs was developed specifically for the purposes of this study; one item was omitted from regression analyses (COVID vaccinated people can affect non-vaccinated people’s health) as we noted that the item could reasonably be interpreted as true (i.e., people vaccinated against COVID-19 can protect the health of others in the community).

## 8. Conclusions

Misbeliefs about COVID-19 vaccines are common in young adults in Australia, with two-thirds of participants harbouring at least one misbelief. Identifying misperceptions and gaps in knowledge about vaccines, such as those found in this study, provides valuable information for public health agencies to better address in vaccine communication initiatives. Additionally, understanding individual factors associated with vaccine misperceptions, including lower health literacy, less knowledge about vaccines, and lower confidence in government and science, can inform targeted public health messaging and the planning of communication strategies for future vaccine rollouts.

## Figures and Tables

**Table 1:** Measures evaluated in this study.

Item	Description and Reference (If Applicable)	Item Scoring and Analysis
**Main outcome**		
**COVID-19 vaccine related misbeliefs** [21,22,23]		Mean value 10 items; scale: 1 = definitely false to 4 = definitely true; 5 = I don’t know enough to make a decision (Responses recoded from 1 (definitely false) to 5 (definitely true); 3 (I don’t know)
	Getting the COVID-19 vaccine gives you COVID-19	
	More people will die of a negative side effect to the COVID-19 vaccine than would actually die from the virus	
	The flu shot provides immunity to COVID-19	
	Supplements are more effective than COVID-19 vaccines	
	COVID vaccines cause immune damage	
	People who have had a COVID-19 vaccine shed the virus to others	
	COVID vaccinated people can affect non-vaccinated people’s health *	
	COVID-19 vaccines have been linked to infertility	
	COVID-19 vaccines contain tracking technology	
	COVID-19 vaccines alter your DNA	
**Explanatory variables**		
**General vaccine knowledge** [24]		Mean value 8 items (0–1): scale: Agree, don’t agree, don’t know (Scored as % correct)
	Vaccines are not needed because diseases can be treated (e.g., with antibiotics)	
	Without broadly applied vaccine programs, smallpox would still exist	
	The effectiveness of vaccines has been proven	
	People would be more resistant to diseases if they were not given so many vaccines	
	Conditions like autism, multiple sclerosis, and diabetes might be triggered through vaccinations	
	The immune system is overloaded if we are given too many vaccinations	
	The doses of chemicals used in vaccines are not dangerous for humans	
	Vaccinations increase the occurrence of allergies	
**Acceptance of vaccine conspiracies** [25]		Mean value 7 items: scale 1 = strongly disagree to 5 = strongly agree
	Data about vaccine safety is often fabricated (made up)	
	People are deceived about the effectiveness of vaccines	
	Immunising is harmful, and this fact is covered up	
	Drug companies cover up the dangers of vaccines	
	Data about vaccine effectiveness is often fabricated (made up)	
	People are deceived about vaccine safety	
	The government is trying to cover up the link between vaccines and autism	
**Perceived risk of COVID-19**		Two individual items, adapted from [26]
	Perceived public threat of COVID-19 (scale: 1 = no threat at all to 10 = very serious public health threat)	
	Concern about getting COVID-19 (scale: 1 = not at all concerned to 4 = very concerned)	
**Confidence in Government**		Mean of 4 items, adapted from [27]; scale: 1 = strongly disagree to 7 = strongly agree
	I am confident in information about COVID-19 vaccines provided by the government	
	I am satisfied with the amount of information about COVID-19 vaccines provided by the government	
	I will follow government advice on COVID-19 vaccination to help protect the wider community	
	I am concerned that government recommendations about COVID-19 vaccines are not safe	
**Trust in Institutions**		Mean of 3 items adapted from [28], scale: 1 = strongly disagree to 7 = strongly agree
	Scientists involved in developing and testing COVID-19 vaccines	
	Researchers involved in trialling COVID-19 vaccine safety and efficacy	
	Medical institutions (GPs, hospitals) involved in distributing COVID-19 vaccines	
**Trust in Government**		Mean of 2 items adapted from [28], scale: 1 = strongly disagree to 7 = strongly agree
	Federal government agencies responsible for managing the control of COVID-19	
	State government agencies responsible for managing the control of COVID-19	
**Confidence in COVID-19 vaccine benefits**		4 items, scale range 1–4, adapted from [29] (reported descriptively)
	Perceived importance of COVID-19 vaccine for health	
	Perceived protection to self and community from getting COVID-19 vaccine	
	Perceived safety of COVID-19 vaccine	
	Perceived concern about having a serious reaction to COVID-19 vaccine	
**Frequency checking COVID-19 information**		
	How often, if at all, do you check social media (such as Facebook or Twitter) for information or updates about COVID-19?	1 item, scale: 1 = Once an hour or more to 5 = I don’t use social media, adapted from [30]
**Exposure to negative information about COVID-19 vaccines**		
	Apart from concerns about rare blood clots, have you seen or heard anything else bad about COVID-19 vaccines? —Yes	Content analysis, adapted from [29]
	In the last week, have you come across any content discouraging people from vaccinating (i.e., on your social media, from friends, at the workplace?) —Yes	

* This item was excluded from regression analyses, as we note that this item could reasonably be interpreted as true (i.e., people vaccinated against COVID-19 can protect the health of others in the community).

**Table 2:** Sample characteristics (N = 2050). Values are shown as n (%) unless otherwise indicated.

Characteristic		n (%)
Age (years), mean (SD)		33.13 (7.9)
Highest level of education	High school or less	460 (22.4%)
	Certificate I-IV	722 (35.2%)
	University	868 (42.3%)
Gender	Woman	1028 (50.1%)
	Man	1009 (49.2%)
	Non-binary, transgender, or prefer not to say ^#^	11 (0.5%)
Residential state or territory	Australian Capital Territory	30 (1.5%)
	Northern Territory	17 (0.8%)
	New South Wales	619 (30.2%)
	Victoria	513 (25.0%)
	Queensland	439 (21.4%)
	Western Australia	214 (10.4%)
	South Australia	159 (7.8%)
	Tasmania	59 (2.9%)
Adequate health literacy		1707 (83.3%)
Digital health literacy (1–5), mean (SD)		3.8 (0.6)
Difficulty finding/understanding information online (1–10), mean (SD)		4.9 (1.9)
How serious of a public health threat do you think COVID-19 is in Australia, (1–10), mean (SD)		7.06 (2.52)
How concerned are you about getting COVID-19?	Not at all concerned	290 (14.1%)
	A little concerned	784 (38.2%)
	Moderately concerned	609 (29.7%)
	Very concerned	367 (17.9%)
Confidence in government (1–7), mean (SD) *		4.52 (1.4)
Trust in institutions (1–7), mean (SD) *		5.06 (1.5)
Trust in government (1–7), mean (SD) *		4.36 (1.7)
Information source about COVID-19 vaccines, %	Official Government source	53%
	Mainstream media	46%
	Social media	45%

* Higher scores indicate greater confidence and trust; ^#^ “non-binary” and “not given” had too few observations and were suppressed.

**Table 3:** Trust in vaccine effectiveness and safety.

How Much Do You Trust *	Effectiveness (n, %)	Safety (n, %)
Childhood vaccines	1760 (85.8)	1801 (87.9)
Flu vaccine	1537 (75.0)	1624 (79.2)
Travel vaccines	1506 (73.5)	1510 (73.7)
Pfizer	1363 (66.5)	1329 (64.9)
AstraZeneca	867 (42.3)	792 (38.6)

* Combined “very much” and “moderately” versus all other responses (“a little” and “not at all”).

**Table 4:** Mean scores (SD) for misperceptions, knowledge, conspiracy.

Variable	n (%)
Vaccine misperceptions (1–5) ^a^	2.2 (0.9)
Vaccine-related conspiracy beliefs (1–5) ^b^	2.6 (0.9)
Vaccine knowledge (% correct) ^c^	49% (26%)

^a^ Responses recoded from 1 (definitely false) to 5 (definitely true); 3 (uncertainty response). ^b^ Higher score indicating more agreement with conspiratorial beliefs. ^c^ Computed based on binary correct/incorrect responses.

**Table 5:** Agreement with COVID-19 vaccine misbeliefs.

	True	False	Don’t Know Enough to Make a Decision
COVID vaccinated people can affect non-vaccinated people’s health *	602 (29.4%)	1081 (52.7%)	367 (17.9%)
People who have had a COVID-19 vaccine shed the virus to others	459 (22.4%)	1185 (57.8%)	406 (19.8%)
COVID-19 vaccines cause immune damage	386 (18.8%)	1172 (57.2%)	492 (24.0%)
COVID-19 vaccines have been linked to infertility	366 (17.9%)	963 (47.0%)	721 (35.2%)
More people will die of a negative side effect to the COVID-19 vaccine than would actually die from the virus	354 (17.3%)	1431 (69.8%)	265 (12.9%)
COVID-19 vaccines alter your DNA	324 (15.8%)	1283 (62.6%)	443 (21.6%)
Getting the COVID-19 vaccine gives you COVID-19	308 (15.0%)	1477 (72.1%)	265 (12.9%)
Supplements are more effective than COVID-19 vaccines	285 (13.9%)	1472 (71.8%)	293 (14.3%)
The flu shot provides immunity to COVID-19	268 (13.1%)	1561 (76.1%)	221 (10.8%)
COVID-19 vaccines contain tracking technology	253 (12.3%)	1488 (72.6%)	309 (15.1%)

* This item was excluded from regression analyses as we note that this item could reasonably be interpreted as true (i.e., people vaccinated against COVID-19 can protect the health of others in the community). We found roughly an equivalent % endorsement between the first and second most common misbeliefs (29% vs. 22% agreement)

**Table 6:** Vaccine-related conspiracy beliefs.

	Agree	Disagree	Unsure
People are deceived about the effectiveness of vaccines	651 (31.8%)	859 (41.9%)	540 (26.3%)
People are deceived about vaccine safety	607 (29.6%)	877 (42.8%)	566 (27.6%)
Drug companies cover up the dangers of vaccines	571 (27.9%)	848 (41.4%)	631 (30.8%)
Data about vaccine safety is often fabricated (made up)	445 (21.7%)	898 (43.8%)	707 (34.5%)
Data about vaccine effectiveness is often fabricated (made up)	434 (21.2%)	994 (48.5%)	622 (30.3%)
The government is trying to cover up the link between vaccines and autism	308 (15.0%)	1105 (53.9%)	637 (31.1%)
Immunising is harmful, and this fact is covered up	280 (13.7%)	1284 (62.6%)	486 (23.7%)

**Table 7:** General vaccine knowledge—Shading indicates correct response.

	Agree	Disagree	Don’t Know
The effectiveness of vaccines has been proven	1629 (79.5%)	212 (10.3%)	209 (10.2%)
Vaccines are not needed because diseases can be treated (e.g., with antibiotics)	235 (11.5%)	1619 (79.0%)	196 (9.6%)
Without broadly applied vaccine programs, smallpox would still exist	1507 (73.5%)	200 (9.8%)	343 (16.7%)
People would be more resistant to diseases if they were not given so many vaccines	384 (18.7%)	1113 (54.3%)	553 (27.0%)
Conditions like autism, multiple sclerosis, and diabetes might be triggered through vaccinations	347 (16.9%)	1015 (49.5%)	688 (33.6%)
The immune system is overloaded if we are given too many vaccinations	473 (23.1%)	904 (44.1%)	673 (32.8%)
The doses of chemicals used in vaccines are not dangerous for humans	829 (40.4%)	504 (24.6%)	717 (35.0%)
Vaccinations increase the occurrence of allergies	351 (17.1%)	753 (36.7%)	946 (46.1%)

**Table 8:** Multivariable truncated linear regression of the misbeliefs score. Higher values of the outcome indicate greater support for misbeliefs. Data are presented as estimated marginal mean differences (95% CIs). Statistical significance is indicated by * *p* < 0.05; ** *p* < 0.01; *** *p* < 0.001. N = 2050 complete cases.

	Main Effect *p*-Value	Base Model (Control Variables Only)	Main Effect *p*-Value	Estimated Marginal Mean Differences (Full Multivariable Model)
**Control Variables**				
Age (/year)		0.008 ** [0.003,0.013]		0.001 [−0.002,0.004]
Male gender (vs. female) ^a^		0.106 ** [0.029,0.183]		−0.013 [−0.066,0.041]
Non-binary, transgender				−0.276 [−0.628,0.076]
Prefer not to say				0.812 [−0.344,1.968]
Education (vs. high school or less)	<0.0001		0.01	
Cert I-IV		0.018 [−0.087,0.123]		−0.018 [−0.091,0.053]
University education		−0.315 *** [−0.416,−0.214]		−0.108 ** [−0.178,−0.038]
State (vs. NSW)	0.087	−0.132 [−0.452,0.188]	0.094	
ACT		−0.120 [−0.444,0.204]		0.063 [−0.157,0.282]
NT		−0.124 [−0.556,0.309]		0.026 [−0.268,0.320]
VIC		0.043 [−0.061,0.146]		0.012 [−0.059,0.083]
QLD		−0.063 [−0.045,0.171]		0.063 [−0.012,0.138]
WA		−0.099 [−0.237,0.037]		−0.049 [−0.145,0.046]
SA		−0.137 [−0.290,0.016]		−0.098 [−0.204,0.008]
TAS		0.0759 [−0.159,0.311]		−0.039 [−0.200,0.120]
Vaccine knowledge				−0.779 *** [−0.912,−0.646]
Health literacy				−0.128 *** [−0.200,−0.056]
Confidence in government				−0.181 *** [−0.241,−0.122]
Conspiratorial beliefs				0.301 *** [0.263,0.340]
Trust in institutions				−0.147 *** [−0.176,−0.117]
Trust in governments				0.016 [−0.007,0.038]
Perceived personal risk of COVID-19				0.033 * [0.0004,0.065]
Frequency checking social media for COVID-19 information or updates				−0.027 * [−0.049,−0.005]

^a^ Marginal mean differences are not reported for gender reported as “not specified” or “other” due to small sample size, but these data were included in the regression model.

**Table 9:** Content discouraging COVID-19 vaccination that participants had witnessed, with frequency of the themes and example quotes.

Theme	n (%)	Example Free Text Response
Negative vaccine side effects and vaccine risk	384 (39.9)	“I heard that getting vaccinated can cause rare blood clots and is dangerous so I choose not to, I don’t want to risk my life”
Conspiracy theories	133 (13.8)	“Tin foil hat people saying the vaccine is designed to make you more likely to catch a virus which will be released in 2025 as the world governments want to cull the population by 6.5 billion people”
Antivax or hesitance towards vaccine	115 (11.9)	“Many people within my community are strongly against the vaccine for many well informed and educated reasons”
Concerns about lack of vaccine testing and contents	75 (7.8)	“The immunization has been rushed way to quickly without adequate testing before it was released to the public”
Distrust in government	64 (6.6)	“Gov’t not reporting the cases of adverse effect and death due to COVID-19 vaccines”

## Data Availability

Data are contained within the article and supplementary material. Any additional data are available on request from the corresponding author.

## Supplementary Materials

The following supporting information can be downloaded at: https://www.mdpi.com/article/10.3390/ijerph19116883/s1, Table S1. Themes identified in free text responses to question “In the last week have you come across any content discouraging people from vaccinating”? (n = 963); Table S2: Sources identified in free text responses to question “In the last week have you come across any content discouraging people from vaccinating”? (n = 963); Table S3: Correlation matrix for study variables.
