# Supplementary material for: COVID-19 Vaccine Misperceptions in a Community Sample of Adults Aged 18–49 Years in Australia
Source: Int J Environ Res Public Health. 2022 Jun 4;19(11):6883. doi: 10.3390/ijerph19116883 (PMC9180736; doi:10.3390/ijerph19116883)
Supplement: Supplementary file 1 [file ijerph-19-06883-s001.zip › ijerph-1732265-supplementary.pdf]

Table S1: Themes identified in free text responses to question “In the last week have you come across any content discouraging people from vaccinating”? (n=963)			
Code	Subcode	n (%)	Example free text response
Vaccine side effects		219 (22.7)	
	General	90 (9.3)	“Side effects from the jab”
	Blood clots	32 (3.3)	“I heard that getting vaccinated can cause rare blood clots & dangerous within some people getting vaccinated so I choose not to at all don't want to risk my life”
	Fertility & Reproductive	24 (2.5)	“That females have had no cycles since getting the vaccination and worried it may cause fertility issues”
	Motor, cognitive, neurological	19 (2.0)	“I have seen a few videos of people who are experiencing neurological symptoms. I have MS and am too scared to take this risk at this point in time. I fear that not enough research has been done to determine what the long term side effects will be”
	AstraZeneca specific	15 (1.6)	“Information discouraging AstraZeneca shared on social media platforms”
	Heart concerns	14 (1.5)	“People have posted about patients being admitted to the hospital with heart conditions after the vaccine although they were healthy beforehand”
	Shaking	9 (0.9)	“I witnessed videos of people experiencing shaking after getting the vaccine.”
	Paralysis	5 (0.5)	“That the vaccine has caused anaphylaxis and partial paralysis”
	Pain	4 (0.4)	“Feeling unusual thing, paining body, not feeling healthy”
	Fever	4 (0.4)	“People having to take time off work after being vaccinated because of the symptoms - bed ridden with chills, fever etc”

	Fainting	3 (0.3)	"People collapsing after having the shot"
Vaccine safety/risk		165 (17.1)	
	Vaccine safety	52 (5.4)	"The vaccines are not safe"
	Vaccine risk	35 (3.6)	"Risk of vaccines"
	Death	78 (8.1)	"Statistics and cases of the vaccine causing more deaths than the actual virus"
Conspiracy		133 (13.8)	
	General	58 (6.0)	"People posting conspiracy theories about the vaccine. Most if it is largely ridiculous."
	Virus legitimacy	40 (4.2)	"Covid is a hoax."
	5G, Tracking, Microchip, Magnetism	24 (2.5)	"Tin foil hat people saying the vaccine is designed to make you more likely to catch a virus which will be released in 2025 as the world governments want to cull the population by 6.5 billion people"
	Vaccine causing disease	11 (1.1)	"Family friend has contracted a disease from one of the vaccines, and has spend most of the last few weeks in and out of hospital."
Antivax/negative or hesitant views about vaccines		115 (11.9)	
	Antivax/negative views about vaccines	109 (11.3)	"Many people within my community are strongly against the vaccine for many well informed and educated reasons"
	Vaccine hesitant	6 (0.6)	"Some vaccine reluctance amongst colleagues."
None (no codable content)		81 (8.4)	"Covid 19 news"
Concern about lack of vaccine testing/contents		75 (7.8)	
	Inadequate vaccine testing	60 (6.2)	"The immunization has been rushed way to quickly without adequate testing before it was released to the public"

	Vaccine ingredients	15 (1.6)	"Vaccines carry unnatural biological components"
Unclear/ Gibberish		73 (7.6)	"A lot of different things."
Distrust in government		64 (6.6)	
	Anti-government sentiment	33 (3.4)	"Gov't not reporting the cases of adverse effect and death due to Covid-19 Vaccines"
	Government control	31 (3.2)	"The government says the vaccines are safe and effective, while changing the recommendations constantly in response to reports of side affects or covid outbreaks, it doesn't instil trust or a sense that the experts know what they're doing, there need to be more openness, informed consent and less propaganda."
Vaccine effectiveness		55 (5.7)	"People saying that some others are still being hospitalised from covid-19 even with being fully vaccinated so what is the point in the vaccine if it's not actually doing it's job"
Broadcasting about/telling others not to vaccinate		52 (5.4)	"People with a large following on Instagram encouraging people to not get vaccinated"
Protest		50 (5.2)	"protests in Sydney and supporting debate on social media"
Uncertainty/unknowns about long term effects of vaccine		39 (4.0)	
	Uncertainty/unknowns about vaccine	15 (1.6)	"Friends and family sharing article's about the uncertainty of the vaccines"
	Long term effects	24 (2.5)	"Not sufficient knowledge about long term effects of Covid vaccines"
Illness post-vaccine		30 (3.1)	"Two people I know who have had the vaccines were unwell for weeks/This friend went into the ICU after getting the vaccine, he nearly died…"

Personal choice/loss of rights/mandates		31 (3.2)	"Being forced to do this is against our constitutional right and is illegal"
Misinformation		26 (2.7)	
	Misinformation	15 (1.6)	"Mainly idiots in Facebook community groups spreading misinformation. I try to avoid reading any news on COVID and prefer to search for research papers to see what science says and not Karen and Bruce from regional VIC"
	Questionable/Alternative evidence about vaccine	11 (1.1)	"People spreading false information in regards to the vaccines on their own social media"
Vaccine roll-out concerns		14 (1.5)	"The waiting time to be fully vaccinated by Astra could be up to 3 months/ 12 weeks, by that time Pfizer would have rolled out."
Natural immunity/vaccine not needed		15 (1.6)	
	Vaccine not needed	12 (1.2)	"People who have respiratory issues or immune deficiencies should get it but for everyone else who is heathy they don't really need it"
	Natural immunity	3 (3.03)	"natural healing is better"
Distrust in vaccine companies		11 (1.1)	"Not to trust the companies behind the vaccines, they are in it purely for profit nothing else"
Fear mongering and hate speech		10 (1.0)	
	Fear mongering	9 (0.9)	"People fear mongering and spreading it to others"
	Hate speech	1 (0.1)	"Protests, tweets, hate speech"
Media manipulation		4 (0.4)	"Fabricated media or non peer reviewed articles"
Health professional suppression		2 (0.2)	"Deaths caused by covid vaccinations. Political gagging of health professionals. Information manipulation by government and media."

Dismissal of adverse reactions		2 (0.2)	"People talking about their experiences of severe reactions that are being swept under the rug"

Table S2: Sources identified in free text responses to question "In the last week have you come across any content discouraging people from vaccinating"? (n=963)

Source code	Subcode	n (%)	Example free text response
Social media		182 (18.9)	
	General	81 (8.4)	"Anti Vax articles on social media"
	Facebook	68 (7.1)	"Any vaccination videos on Facebook listing negative side effects"
	Instagram	17 (1.8)	"Instagram posts shared by friends listing views of non vax posts"
	TikTok	9 (0.9)	"People talking about the dangers of the vaccine on TikTok"
	Youtube	4 (0.4)	"YouTube on Questioning the Effectiveness of Vaccine"
	Twitter	2 (0.2)	"People on Facebook and Twitter and Instagram questioning the safety of vaccines"
	Reddit	1 (0.1)	"On Reddit - posts questioning the safety of the vaccines as they were so rushed."
Family/Friends/ Colleague		57 (5.9)	"A friend that does not believe he should get vaccinated to keep others safe"
Traditional Media (News, TV, Print, Flyers)		46 (4.8)	"a headline on the news"
"Video"/"Picture"/"Post"/"Article" but no further information		27 (2.8)	"I witnessed videos of people experiencing shaking after getting the vaccine."
Protest		21 (2.2)	"People protesting against lockdowns etc"
In person/physical public location		7 (0.7)	"A customer came in claiming it was nonsense they're making us live like this and have to get stabbed with random shit they mixed together (or something along those lines)"
Healthcare worker/scientist		7 (0.7)	"local chemist stating he would not be getting and would not be giving it to his children, footage of a woman strapped to a bed with a severe reaction to immunisation, lady in Sydney who died after receiving her vaccine"
Public figure		7 (0.7)	"Pauline Hanson explaining that the vaccines have been developed at such a fast rate, it must have problems with them."
Community Group		3 (0.3)	"Many people within my community are strongly against the vaccine for many well informed and educated reasons"

Table S3. Correlation matrix for study variables.

	Age	Gender	State	Personal Risk	Australia Risk	Knowledge	Misbeliefs	Conspiracy	General Confidence	Confidence in Govt	Trust in Institutions	Trust in Govt	Education
Age	1												
Gender	0.0276	1											
State	0.0523*	-0.0813*	1										
Personal Risk	0.0168	-0.0305	-0.0982*	1									
Australia Risk	0.0371	0.0174	-0.1616*	0.5145*	1								
Knowledge	-0.0628*	-0.0189	0.0094	0.2740*	0.0961*	1							
Misbeliefs	0.0615*	0.0454*	-0.0125	-0.3339*	-0.1362*	-0.5835*	1						
Conspiracy	0.0727*	0.1009*	-0.0166	-0.3062*	-0.0787*	-0.6001*	0.6564*	1					
General Confidence	-0.0444*	-0.0136	-0.1290*	0.5012*	0.4875*	0.3882*	-0.4609*	-0.3743*	1				
Confidence in Govt	-0.1111*	0.0131	-0.0448*	0.3422*	0.2567*	0.3225*	-0.4253*	-0.3393*	0.538*	1			
Trust in Institutions	-0.1136*	-0.0034	-0.0298	0.4052*	0.2533*	0.5277*	-0.6153*	-0.582*	0.6026*	0.5657*	1		
Trust in Govt	-0.1048*	0.0125	-0.018	0.3323*	0.2539*	0.3452*	-0.4036*	-0.3556*	0.5395*	0.6191*	0.6959*	1	
Education	0.1325*	0.0710*	-0.0803*	0.0900*	0.1269*	0.0952*	-0.1339*	-0.0513*	0.1599*	0.1382*	0.1533*	0.1210*	1
